# Mdm2 Induces Mono-Ubiquitination of FOXO4

**DOI:** 10.1371/journal.pone.0002819

**Published:** 2008-07-30

**Authors:** Arjan B. Brenkman, Peter L. J. de Keizer, Niels J. F. van den Broek, A. G. Jochemsen, Boudewijn M. Th. Burgering

**Affiliations:** 1 Department of Physiological Chemistry and Centre for Biomedical Genetics, University Medical Centre Utrecht, Utrecht, The Netherlands; 2 Department of Molecular Cell Biology, Leiden University Medical Center, Leiden, The Netherlands; 3 Department of Physiological Chemistry and Centre for Biomedical Genetics Universiteitsweg, Utrecht, The Netherlands; National Institutes of Health, United States of America

## Abstract

**Background:**

The Forkhead box O (FOXO) class of transcription factors are involved in the regulation of several cellular responses including cell cycle progression and apoptosis. Furthermore, in model organisms FOXOs act as tumor suppressors and affect aging. Previously, we noted that FOXOs and p53 are remarkably similar within their spectrum of regulatory proteins [1]. For example, the de-ubiquitinating enzyme USP7 removes ubiquitin from both FOXO and p53. However, Skp2 has been identified as E3 ligase for FOXO1, whereas Mdm2 is the prime E3 ligase for p53.

**Principal Findings/Methodology:**

Here we provide evidence that Mdm2 acts as an E3 ligase for FOXO as well. *In vitro* incubation of Mdm2 and FOXO results in ATP-dependent (multi)mono-ubiquitination of FOXO similar to p53. Furthermore, *in vivo* co-expression of Mdm2 and FOXO induces FOXO mono-ubiquitination and consistent with this result, siRNA-mediated depletion of Mdm2 inhibits mono-ubiquitination of FOXO induced by hydrogen peroxide. Regulation of FOXO ubiquitination by Mdm2 is likely to be direct since Mdm2 and FOXO co-immunoprecipitate. In addition, Mdm2-mediated ubiquitination regulates FOXO transcriptional activity.

**Conclusions/Significance:**

These data identify Mdm2 as a novel E3 ligase for FOXOs and extend the analogous mode of regulation between FOXO and p53.

## Introduction

Forkhead box O (FOXO) transcription factors have recently gained considerable attention because of their potentially critical role in aging [1], [2]. The paradigm in this respect is the *C. elegans* FOXO ortholog DAF-16. Lifespan extension through a number of genetic and non-genetic interventions in these nematodes requires at least in part DAF-16 [2]. Especially, the effects of lowered insulin signaling critically depend on DAF-16 and DAF-16 acts downstream of the insulin signaling pathway consisting of the lipid kinase phosphoinositide-3 kinase (PI-3K) and the serine/threonine protein kinase B (PKB/AKT). PKB directly phosphorylates DAF-16/FOXO and this results in nuclear exclusion and therefore reduced DAF-16/FOXO transcriptional activity [3], [4].

Aging may also result from the accumulating damage caused by reactive oxygen species (ROS)[5]. In this respect regulation of cellular anti-oxidant capacity by DAF-16/FOXO provided rationale for its effect on lifespan. Interestingly, FOXO itself is also regulated by ROS and treatment of cells with hydrogen peroxide, which increases cellular oxidative stress, results in nuclear translocation of FOXO [6], [7]. FOXOs are regulated through a multitude of post-translational modifications (PTMs) including phosphorylation, acetylation and ubiquitination (reviewed in [1]). Whereas PKB-mediated phosphorylation results in exclusion of FOXO from the nucleus, the mechanism and/or PTMs responsible for relocalization to the nucleus after increased cellular oxidative stress, remain poorly understood. However, the enzymes responsible for adding these modifications are remarkably similar between p53 and FOXO (for a discussion see [1]). With respect to the regulation of ubiquitination we previously identified USP7 as a de-ubiquitinating enzyme for FOXO4 [8] and USP7 is also a de-ubiquitinating enzyme for p53 [9]. FOXOs are relatively stable proteins with a half-life of approximately 8–10 hrs in untransformed cells [8]. In transformed/oncogenic cells, especially cells transformed through activation of PI-3K signaling, FOXO protein half-life is shortened [10]–[12]. This is likely due to PI-3K/PKB mediated upregulation of Skp2 in these cells, as Skp2 has been identified as an ubiquitin E3 ligase responsible for FOXO poly-ubiquitination and degradation [10]. Consistent with Skp2 regulation by PKB and FOXO being degraded in a Skp2-dependent manner, several other PKB targets have been reported to be degraded in a Skp2-dependent manner as well [11], [13]. Previously, we demonstrated that the signaling function of FOXO4 is regulated by mono-ubiquitination especially after increased cellular oxidative stress [8]. Mono-ubiquitination correlates with increased nuclear localization of FOXO and hence increased transcriptional activity. Consequently, USP7 expression inhibited FOXO4 transcriptional activity due to de-ubiquitination of FOXO4 and re-localization to the cytosol. To further understand the regulation of mono-ubiquitination of FOXOs we searched for ubiquitin E3 ligases that would ligate ubiquitin onto FOXO4. Here, we report the identification of Mdm2 as an E3 ligase for FOXO4 that mediates mono-ubiquitination of FOXO4 after increased cellular oxidative stress.

## Results

**Figure 1:**
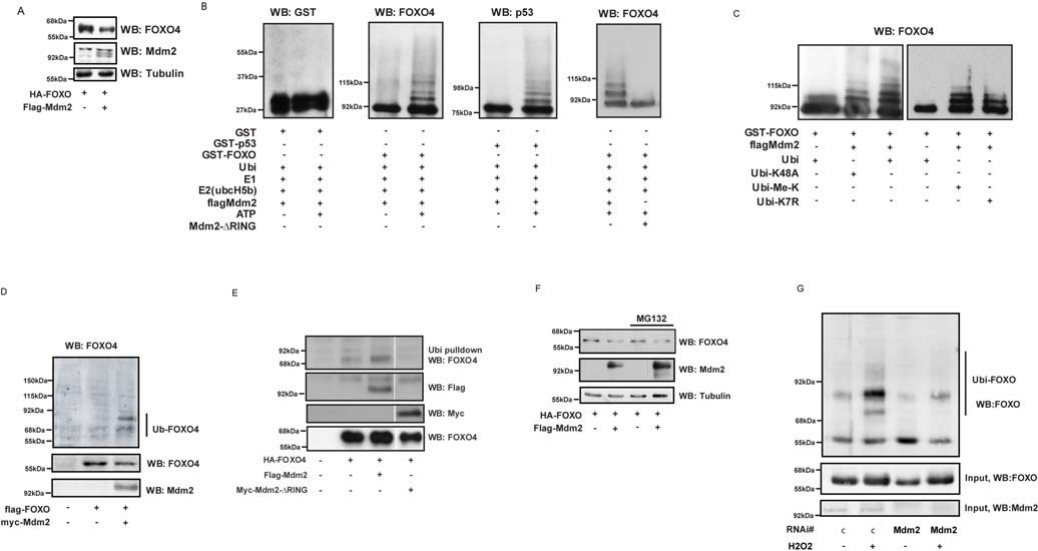
FOXO4 is a substrate for Mdm2 ubiquitination. (a) Mdm2 co-expression decreases FOXO protein levels. FOXO4 and Mdm2 were co-expressed in HEK293T cells. Cell lysates were probed by western blot analysis as indicated. (b) Mdm2 ubiquitinates FOXO4 and p53 *in vitro* with similar stoichiometry. Purified Mdm2 or Mdm2-delta-RING was incubated with GST-FOXO4, GST-p53 or GST alone, together with Ubiquitin and E1-E2-*in vitro* recombinant proteins. Ubiquitination was measured 2 h., after addition of rATP. (c) FOXO4 is multi-mono-ubiquitinated by Mdm2. The experiment was performed as in (b), using ubiquitin proteins (Ubi-K48A, Methylated Ubiquitin and Ubi-K7R) that are unable to poly-ubiquitinate. (d) Mdm2 ubiquitination of FOXO *in vivo*. HEK293T cells were transfected with myc-Mdm2, Flag-FOXO4 or control vector together with His-Ubiquitin. After 24 h, cells were treated with 50 µM H_2_O_2_ for 15 min, and subjected to a ubiquitination assay (see Methods) (e) FOXO4 mono-ubiquitination *in vivo* depends on the Ring finger of Mdm2. HEK293T cells were transfected with indicated constructs and subjected to a ubiquitination assay. (f) Mdm2 mediated FOXO4 downregulation is MG132 insensitive. MCF7 cells were transfected with the indicated constructs and treated with MG132 o/n. (g) FOXO4 mono-ubiquitination is dependent on Mdm2. HEK293T cells were treated with either control (c) or human Mdm2 RNAi and subsequently transfected with His-Ubiquitin and HA-FOXO. Cells were treated with 50 µM H_2_O_2_ for 15 min and subjected to a ubiquitination assay.

In our attempt to identify potential E3 ligases for FOXOs we co-expressed several candidate E3 ligases with FOXO and noticed that Mdm2 co-expression resulted in an apparent reduction in FOXO4 expression (**Fig. 1a**). In transient expression experiments reduced protein expression can occur through various mechanisms including promoter squelching. However, as Mdm2 induces ubiquitin-mediated breakdown of target proteins we first analyzed whether Mdm2 could catalyze ubiquitin addition to FOXO4 *in vitro*. To this end we reconstituted a functional E1-E2-Mdm2 ubiquitin ligase system using purified proteins and added GST-FOXO4 as a substrate. Only in the presence of rATP this resulted in the addition of multiple ubiquitin moieties to GST-FOXO causing a laddering indicative for poly-ubiquitination or multiple mono-ubiquitination (**Fig. 1b**). Importantly, this *in vitro* reconstituted system displayed similar activity towards GST-p53, but not to GST alone, suggesting that *in vitro* FOXO is as good a substrate for Mdm2 as is p53. Mdm2 uses a C-terminal RING finger domain, critical to its function as an E3-Ligase. A Mdm2 mutant lacking this domain was tested and found unable to ubiquitinate FOXO4 (**Fig. 1b**), highlighting the specificity of Mdm2 and the dependency on its E3-ligase activity to mono-ubiquitinate FOXO4 *in vitro*. To further address whether the *in vitro* observed laddering represents poly-ubiquitination or multiple mono-ubiquitination, a number of ubiquitin mutants instead of wild-type ubiquitin were analyzed in the *in vitro* ubiquitination assay. Using ubiquitin-K48A, defective in K48-mediated ubiquitin branching which targets proteins for the proteasome, and ubiquitin-K7R and methyl-ubiquitin both defective in mediating poly-ubiquitination all resulted in same pattern of mdm2-mediated GST-FOXO laddering (**Fig 1c**). Taken together these results clearly show that in an *in vitro* reconstituted system Mdm2 can act as an E3-ligase for FOXO4 and that in contrast to what has been reported for p53, Mdm2 catalyzes multiple mono-ubiquitination of GST-FOXO4 rather than poly-ubiquitination.

Next, we tested whether Mdm2 also can ubiquitinate FOXO4 *in vivo*. Co-expression of flag-FOXO4 and myc-Mdm2 induced mono-ubiquitination of FOXO4 (**Fig. 1d**). We did not observe substantial poly-ubiquitination, also not in the presence of the proteasome inhibitor MG132 (data not shown). Also, the delta-RING domain Mdm2 mutant did not induce FOXO4 mono-ubiquitination (**Fig 1e**). Albeit consistent with our *in vitro* data this questions the mechanism underlying the reduced detection of FOXO4 protein by immunoblotting after overexpression of Mdm2. Reduced detection of FOXO4 concomitant with Mdm2 overexpression would normally be taken to indicate proteasomal degradation of FOXO4 and this should be reversed by MG132 treatment. However, we did not observe substantial rescue of FOXO4 protein expression after MG132 treatment, despite observing accumulation of auto-poly-ubiquitinated Mdm2 species, which indicates that the MG132 treatment did work (**Fig. 1f**). Again this is consistent with the observed lack of poly-ubiquitination and suggests that either FOXO4 is degraded through another pathway for example caspase-mediated breakdown, or alternatively, that (multiple) mono-ubiquitinated FOXO4 is targeted to a cellular compartment for example PML bodies from which it is not efficiently extracted. To confirm our MG132 experiments, we performed FOXO4 half-life studies. Transfection of Mdm2 did not affect the half-life of co-transfected FOXO4 (Supplementary **Fig. S1**). Taken together these results indicate that Mdm2 expression does not lead to FOXO4 degradation by means of regulating its protein stability through the proteasome. This finding is consistent with our MG132 experiments. Thus in all approaches we come to the conclusion that Mdm2 does not substantially affect FOXO4 protein half-life.

Finally, to further substantiate a role for endogenous Mdm2 in regulating the ubiquitin status of FOXO4 *in vivo*, we used siRNA against the human ortholog of Mdm2 (Supplementary **Fig. S2**). As reported previously, increasing cellular oxidative stress by treating cells with hydrogen peroxide induced mono-ubiquitination of both FOXO4 and FOXO3a [8] and Supplementary **Fig. S3**. Importantly, siRNA-mediated knockdown of Mdm2 significantly reduced hydrogen peroxide-induced mono-ubiquitination of FOXO4 (**Fig. 1h**). Together, these data provide compelling evidence that Mdm2 can mediate FOXO4 mono-ubiquitination *in vitro* and *in vivo*.

**Figure 2:**
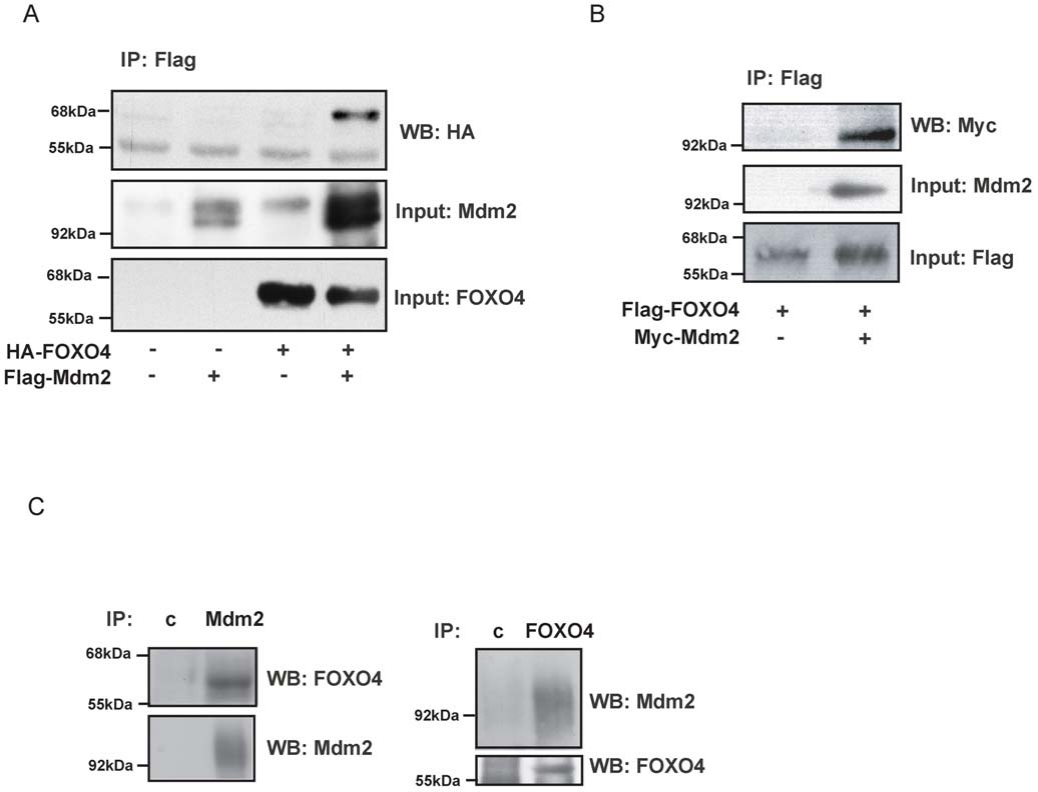
Mdm2 interacts with FOXO4. (a) HA-FOXO4 and Flag-Mdm2 were co-expressed in HEK293T cells, co-immunoprecipitated for Flag and probed as indicated. (b) HEK293T cells were transfected with Flag-FOXO4 and Myc-Mdm2. Lysates were co-immunoprecipitated with Flag antibody and proteins were detected as indicated. (c) FOXO4 and Mdm2 interact *in vivo*. HEK293T cells were immunoprecipitated for FOXO4, Mdm2 or isotype controls (c) and probed as indicated. Prior to Co-Ip, cells were treated for 15 min. with 200 µM hydrogen peroxide.

**Figure 3:**
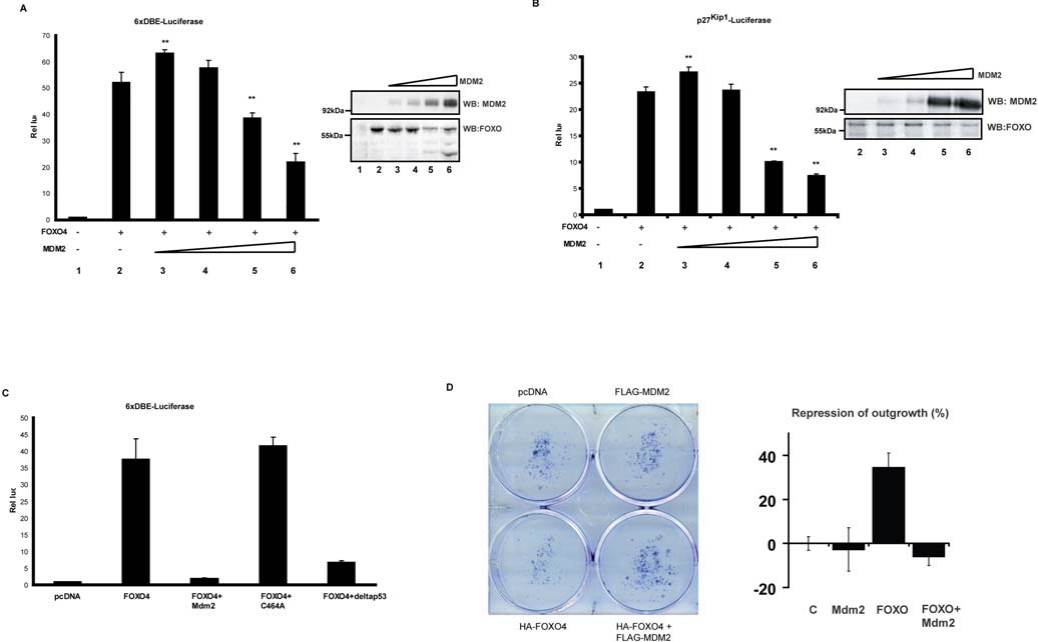
Mdm2 regulates FOXO4 activity. (a, b) Mdm2 regulates FOXO activity in a bell-shaped manner. MCF7 cells were transfected with indicated constructs, TK-Renilla and either a luciferase construct with 6 perfect FOXO4 DNA binding elements (6xDBE) (a) or a luciferase construct under the control of the endogenous p27^kip1^ promoter (b). Representative data are shown as mean±s.d. of triplicates. The significance of changes in the lanes with Mdm2 as compared to wild-type FOXO (lane 2) was confirmed by t-test (**p<0.005). (c) FOXO regulation of Mdm2 is independent of p53 but dependent on its RING finger activity. Luciferase activity was measured in MCF7 cells, 24 h after transfection with indicated constructs, TK-Renilla and (6xDBE). (d) FOXO mediated cell cycle arrest is blocked by Mdm2. A14 cells were transfected by indicated constructs together with pbabePuro. Colony outgrowth of puromycin selected cells was monitored after 10 days.

To test the possibility that Mdm2 could directly regulate FOXO we analyzed binding between Mdm2 and FOXO4. Upon co-expression of Flag-Mdm2 and HA-FOXO4, FOXO4 was co-immunoprecipitated with Mdm2, and *vice-versa* (**Fig. 2a,b**). Consistent with our *in vitro* data, this result suggests that Mdm2 directly binds and regulates FOXO4, rather than through regulating the activity of de-ubiquitinating enzymes such as USP7. Next, we tested binding between endogenous FOXO4 and Mdm2 proteins and observed reciprocal co-immunoprecipitation between endogenous FOXO4 and Mdm2 in HEK293T cells (**Fig 2c**). Mono-ubiquitination of FOXO4 results in increased transcriptional activity of FOXO4 [8] and this is reversed by USP7-mediated de-ubiquitination. To see whether Mdm2 would also regulate FOXO4 transcriptional activity we performed FOXO reporter assays. Expression of increasing amounts of Mdm2 resulted in a bell-shaped regulation of FOXO4 transcriptional activity. Low amounts of Mdm2 transfected induced a reproducible increase in FOXO4 transcriptional activity on two different FOXO responsive reporters (**Fig. 3a** (6xDBE-luciferase) and **Fig. 3b** (p27-luciferase)), consistent with the notion that Mdm2 can induce mono-ubiquitination of FOXO4. In contrast, higher amounts of Mdm2 transfected resulted in reduced FOXO4 transcriptional activity. This suggests that the multiple mono-ubiquitination of FOXO4 induced by Mdm2 *in vitro* and possibly *in vivo* by high levels of Mdm2 represents inactivation of FOXO4. In effect this would be similar to poly-ubiquitination and degradation, but in contrast this leaves FOXO4 to be re-activated by de-ubiquitination by USP7 [8]. To assess the structural requirements of Mdm2 to regulate FOXO4 transcriptional activity we compared the effect of wild-type Mdm2 on FOXO4 transcriptional activity with that of Mdm2 mutated in its RING domain, and with that of Mdm2 mutated in its p53 interaction domain (**Fig. 3c**). The RING domain mutant of Mdm2 did not affect FOXO4 transcriptional activity indicating that Mdm2 regulated FOXO4 activity requires a functional E3 ligase domain. In contrast, p53 binding to Mdm2 appears not involved as Mdm2 defective in binding to p53 regulated FOXO4 in a manner identical to wild-type Mdm2. Ectopic expression of FOXO4 in cells induces cell cycle arrest and induction of apoptosis [1] and this can be monitored by a reduction in colony formation ([14] and **Fig 3d**). Similar to decreased transcriptional activity at higher levels of Mdm2 co-expression Mdm2 represses the ability of FOXO4 to inhibit colony formation.

## Discussion

Here we provide evidence that Mdm2 is an E3-ligase that can ligate ubiquitin onto FOXO4 both *in vitro* and *in vivo*. Mono-ubiquitination of FOXO4 as induced by hydrogen peroxide treatment of cells requires endogenous Mdm2, because siRNA mediated knockdown of Mdm2 prevents mono-ubiquitination. This shows that Mdm2 is at least functional in regulating mono-ubiquitination of FOXO4. *In vitro*, Mdm2 induces a pattern of ubiquitination that normally is considered indicative of poly-ubiquitination. However, alternative to poly-ubiquitination, extensive mono-ubiquitination on multiple different lysine residues, 19 of which are present in FOXO4, can cause a similar characteristic laddering. This would be consistent with the current view that low mobility species of ubiquitinated p53 represent mono-ubiquitinated p53 (Fig. 1 and [15], [16]. To further discriminate between these possibilities we used a number of ubiquitin mutants that are defective in poly-ubiquitination (K48A, K7R and methyl-ubiquitin).

When these mutants were used as only ubiquitin donor in the *in vitro* assay, Mdm2 catalyzed a highly similar, if not identical, pattern of laddering compared to including wild-type ubiquitin in this assay. This strongly suggests that *in vitro* Mdm2 catalyzes preferentially only (multi-)mono-ubiquitination of GST-FOXO4. Also *in vivo* overexpression or siRNA-mediated knockdown of Mdm2 resulted in the induction or loss of FOXO4 mono-ubiqitination respectively. Thus we conclude that Mdm2 regulates mono-ubiquitination of FOXO4. However, we observed an apparent reduction in protein expression of FOXO4, especially after high expression of Mdm2 and this would suggest protein degradation most likely through poly-ubiquitination-mediated proteasomal degradation. This would be at odds with the conclusion that Mdm2 catalyzes mono-ubiquitination. To establish whether proteasomal degradation is causal to the reduced detection of HA-FOXO4 after flag-Mdm2 overexpression we treated cells with MG132. Inhibition of proteasome-mediated degradation did not result in increased detection of HA-FOXO4 despite observed accumulation of Mdm2. Thus, this result does not implicate Mdm2 induced poly-ubiquitination of HA-FOXO4 and subsequent degradation through the proteasome and is consistent with the lack of effect of MG132 treatment on FOXO4 ubiquitination *in vivo* (Fig 1, and B.M.T.B, unpublished data). Consistent with these observations, protein stability experiments indicate that Mdm2 does not affect FOXO4 protein stability. Consequently, reduced detection of FOXO4 after Mdm2 overexpression is likely due to other mechanism(s). This could be alternative mechanism(s) of degradation such as lysosomal degradation or protease-mediated degradation (e.g. caspase). Alternatively, mono-ubiquitination has been shown in several cases to regulate cellular localization of proteins and thus the apparent reduction in protein expression may equally represent a shift of target protein into a complex or towards a cellular location that results in inefficient extraction of protein and thereby reduced detection after immunoblotting. Indeed, recent developments have provided multiple examples in different signaling pathways that ubiquitination serves other purposes than merely targeting proteins for degradation [17]–[19]. This raises the interesting possibility that the initial function of (mono-)-ubiquitination is to provide a means to regulate protein function similar to for example phosphorylation. However, to terminate the signaling function of (mono-)ubiquitination a cell can choose between either de-ubiquitination or poly-ubiquitination. Depending on the urgency to terminate signaling, poly-ubiquitination and subsequent degradation, may be the preferred mode.

While our study was in progress Yang et al. also reported ubiquitination of FOXO3a by Mdm2 [20]. However, in contrast to our results presented here, their study suggests a role for Mdm2 mainly in the breakdown of FOXO3a. As discussed above, in our experiments only high expression of Mdm2 may result in induced breakdown of FOXO4.

Yang et al. also implicated a role for ERK in the regulation of FOXO3a by Mdm2 and provide evidence that FOXO3a phosphorylation by ERK through an unknown mechanism induces Mdm2 binding to FOXO3a [20]. Importantly, Yang et al. use EGF as stimulus whereas we use peroxide stress. It is of importance to note that the stimuli used by others (EGF, PDGF, insulin) all inhibit FOXO function whereas we and others have shown that oxidative stress (e.g. peroxide) as used here activates FOXOs [6]. This is an essential difference. In addition, for PDGF and insulin stimulation several previous studies have shown that Skp2 is involved in degradation of FOXO induced by these factors [10], [11]. In addition, with respect to the issue here, several interesting observations within these studies were made. Firstly, FOXO half-life in ‘normal’ cells is around 8–10 hrs similar to our previous observations ([8] and the results in this study). Second, only in cells transformed through PI3K activation (v-Ha-RAS, Active PI3K alleles) FOXO half-life is shortened [21], but again this is in these studies a PKB/AKT and Skp2 mediated process (and not ERK-Mdm2). We are tempted to speculate that Mdm2 induces FOXO mono-ubiquitination; this results in activation of FOXO. Activation can be terminated by USP7 de-ubiquitination or alternatively by Skp2-mediated poly-ubiquitination and degradation. The latter occurs as a result of oncogenic transformation through PI3K/PKB/AKT, but possibly also through ERK signalling. Thus if one considers the possibility of Mdm2 being a ‘priming’ E3-ligase for FOXO and Skp2 the branching E3-ligase, these different results can be reconciled. Clearly further studies are required to fully appreciate the role of ubiquitination in FOXO regulation in response to various cellular conditions.

Mono-ubiquitination is observed and studied thus far, for proteins with a relative long half-life, such as PTEN [22], EGF receptor [23] and FOXOs (approximately 10 hrs in untransformed cells [8]). In contrast, mono-ubiquitination has not yet been considered for short lived proteins such as cell cycle regulators (cyclins) and oncogenes (myc, beta-catenin). However, establishing mono-ubiquitination for these short-lived proteins may just be a technical challenge. Indeed, recent results with p53 may provide basis for such a paradigm shift. Whereas, initially p53 served as a classical example of a protein regulated through protein degradation, it is by now clear that mono-ubiquitination of p53 occurs and serves to provide a new signaling function to p53 [15], [24]. Instead of acting as a transcription factor, mono-ubiquitination of p53 serves as a signal to relocate p53 from the nucleus to mitochondrial membrane [24]. Along the same line of reasoning, the role of Mdm2 in ubiquitination of p53 is now being discussed [17], [25], [26]. Thus, the possibility is being raised that endogenously expressed Mdm2 is actually preferentially involved in mono-ubiquitination of p53, whereas aberrant high expression of Mdm2 may result in poly-ubiquitination and degradation of p53. Essentially, our observations presented here indeed fully support a role for Mdm2, at endogenous level, in regulating mono-ubiquitination of in this case FOXO4.

In summary, we have identified Mdm2 as an ubiquitin E3 ligase for FOXO4 which functions in oxidative stress-induced FOXO4 mono-ubiquitination. This extends the network of co-regulatory proteins of FOXO and p53 and therefore supports a model of co-evolution of stress maintenance mechanisms.

## Materials and Methods

### Cell culture and Transfection

HEK293T, MCF7 and A14 cells (3T3 fibroblasts stably expressing the insulin receptor) were maintained in Dulbecco's Modified Eagle medium (Cambrex), supplemented with 10% fetal bovine serum, penicillin/streptomycin and 0.05% glutamine. Transient transfections were performed with FuGENE6 (Roche). Cycloheximide experiments were performed as described [8]

### Constructs, antibodies and RNAi

pMT2-HA-FOXO4 and pMT2-Flag-FOXO4, His-Ubiquitin, Flag-Mdm2, the inactive Mdm2 RING-finger mutant C464A, Myc-Mdm2-delta-RING domain, MycMDM2-delta-p53 mutants and Myc-Mdm2 have been described previously [8], [27]. The luciferase constructs TK-Renilla, 6xDBE and the p27^kip1^ Luciferase promoters have been described [6]. Non-targeting RNAi duplex (c), RNAi smartpool oligonucleotides specific for human Mdm2 were purchased from Dharmacon. Cells were transfected with 20 µM RNAi with Oligofectamine (Invitrogen) for RNAi oligonucleotides and FuGENE6 for DNA constructs. DNA constructs were transfected 8 h. after the last RNAi oligonucleotide transfection. The antibodies against FOXO4 (834) and HA (12CA5), have been described [4]. The following antibodies were purchased; Mdm2 (SMP-14, Santa Cruz), Tubulin and Flag-M2 (Sigma).

### Co-immunoprecipitation assay

For co-immunoprecipitation studies, 50 µl Protein-A Sepharose beads were pre-coupled to 1 µg of the indicated antibody. Cells were lysed in Co-IP buffer (20 mM Tris-HCl pH 8.0, 1% NP-40, 10% glycerol, 1 mM MgCl_2_, 1 mM EDTA, 150 mM NaCl, protease and phosphatase inhibitors) and incubated as described previously [28]. For endogenous co-immunoprecipitations, cells were treated prior to lysis with hydrogen peroxide (200 µM, 15 min.).

### Luciferase Reporter assay

Cells were transfected with the indicated constructs plus 20 ng TK-Renilla per condition as a transfection efficiency control. Lysates were measured after 24 h by the Promega Dual Luciferase reporter assay.

### Ubiquitination assays

The *in vitro* ubiquitination assay was performed essentially as described [29]. Flag-Mdm2 was purified from HEK293T cells with Flag-M2 beads (Sigma). Precipitated protein was washed with RIPA, and eluted off with Flag peptide (Sigma). Eluted protein was dialysed o/n with a buffer containing (25 mM HEPES-pH 8.0, 150 mM NaCl, protease inhibitors). The *in vitro* ubiquitination assay was initiated with the addition of ATP (f.c.2.5 mM) and quenched after 2 h with Laemmli Sample Buffer. Purified GST and GST-FOXO4 were a kind gift from H. de Ruiter. Purified GST-p53, E1, E2 (UbcH5b) were kind gifts from Dr. K.W. Mulder. Ubiquitin, Ubiquitin-K7R (All lysines mutated to arginine), Ubiquitin-K48A (lysine 48 mutated to Alanine) and methylated Ubiquitin (all lysines methylated) were purchased at Boston Biochem. *In vivo* ubiquitination assays were essentially performed as described [8]. In brief, HEK293T cells were transfected with the indicated constructs. 48 h. post transfection, cells were left untreated or treated as indicated, and lysed in lysis buffer (8 M Urea, 10 mM Tris-HCl pH 8.0, 100 mM Na_2_HPO_4_/NaH_2_PO_4_, 0.2% TX-100, 5 mM NEM, protease inhibitors). Ubiquitinated proteins were precipitated using Ni-NTA agarose beads and analysed by WB.

### Colony outgrowth assay

Equal amounts of A14 cells were plated in triplicate in 6-well dishes and transfected with 2 µg of the indicated constructs in combination with 0.5 µg pbabe-puro. 24 hours post-transfection cells were placed under selection with 2 µg/ml Puromycin. Every two days the selection medium was refreshed. At 10 days post transfection cells were fixed for 10 minutes with ice-cold methanol and colonies were stained with 0.5% crystal violet, dissolved in 25% methanol. The plates were washed with dH_2_O and dried overnight.

## Supporting Information

Figure S1 FOXO4 protein stability is not affected by Mdm2. MCF7 were transfected with either FOXO4 alone, or in combination with Mdm2. Transfected cells were treated with cycloheximide (CHX) for indicated times. Relative protein expression levels were quantified and displayed in a graph (bottom)
(0.88 MB TIF)

Figure S2 Efficient knockdown of human Mdm2 by RNAi.
(0.79 MB TIF)

Figure S3 Mono-ubiquitination of FOXO4 and FOXO3a is induced upon peroxide stress. HEK293T cells were transfected with indicated constructs and His-Ubiquitin. Cells were left untreated or were treated with 50 µM H2O2 for 30 min, lysed and subjected to a ubiquitination assay. (*) Ubiquitinated FOXO4, (**) Ubiquitinated FOXO3a.
(1.21 MB TIF)
