# Supplementary figures and images for: Mdm2 Induces Mono-Ubiquitination of FOXO4
Source: PLoS One. 2008 Jul 30;3(7):e2819. doi: 10.1371/journal.pone.0002819 (PMC2475507; doi:10.1371/journal.pone.0002819)

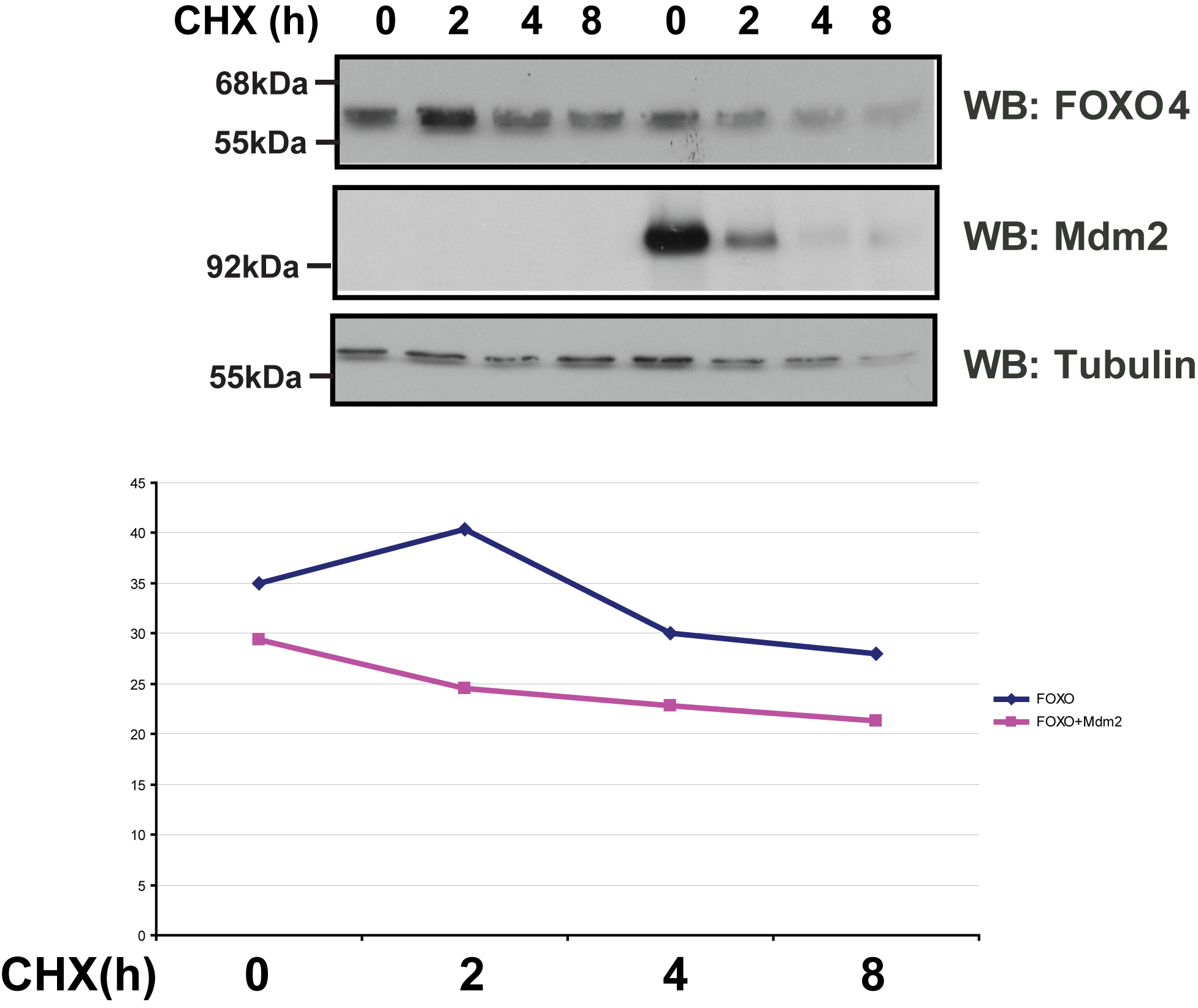

Supplement: Figure S1 — FOXO4 protein stability is not affected by Mdm2. MCF7 were transfected with either FOXO4 alone, or in combination with Mdm2. Transfected cells were treated with cycloheximide (CHX) for indicated times. Relative protein expression levels were quantified and displayed in a graph (bottom) (0.88 MB TIF) [file pone.0002819.s001.tif]

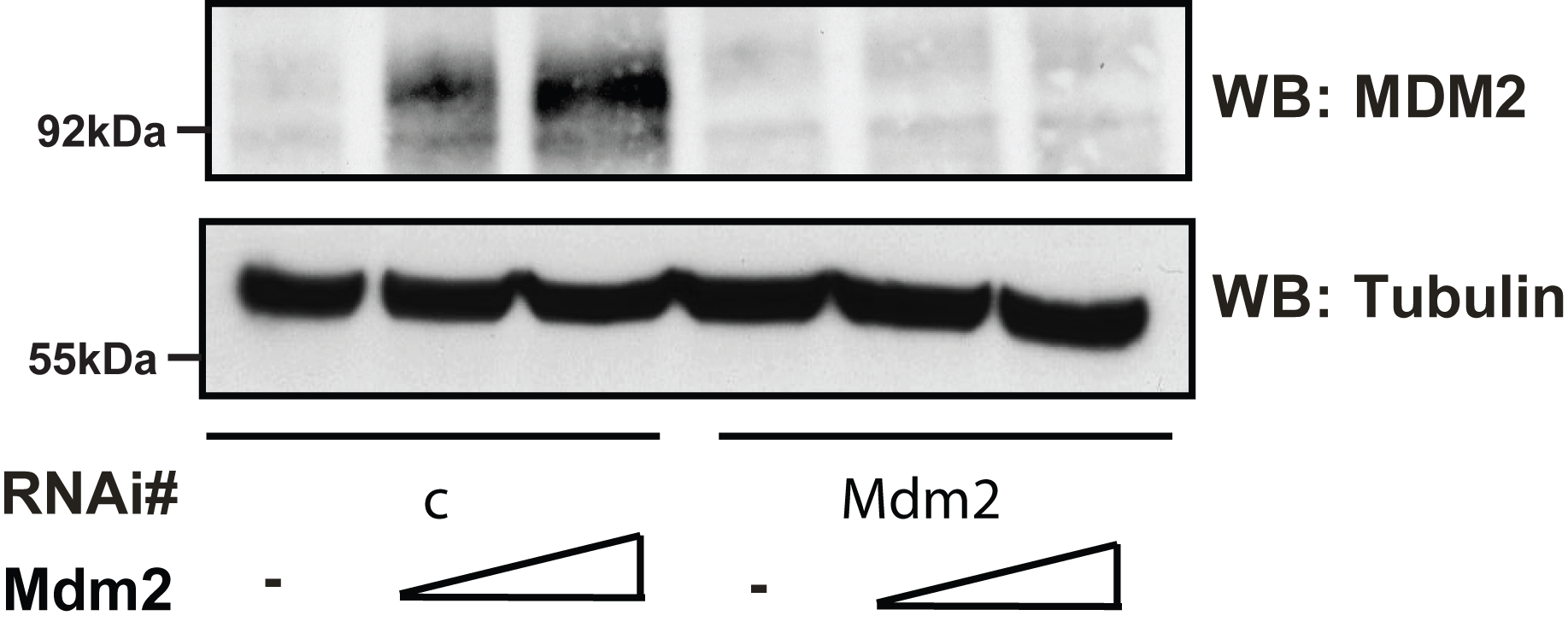

Supplement: Figure S2 — Efficient knockdown of human Mdm2 by RNAi. (0.79 MB TIF) [file pone.0002819.s002.tif]

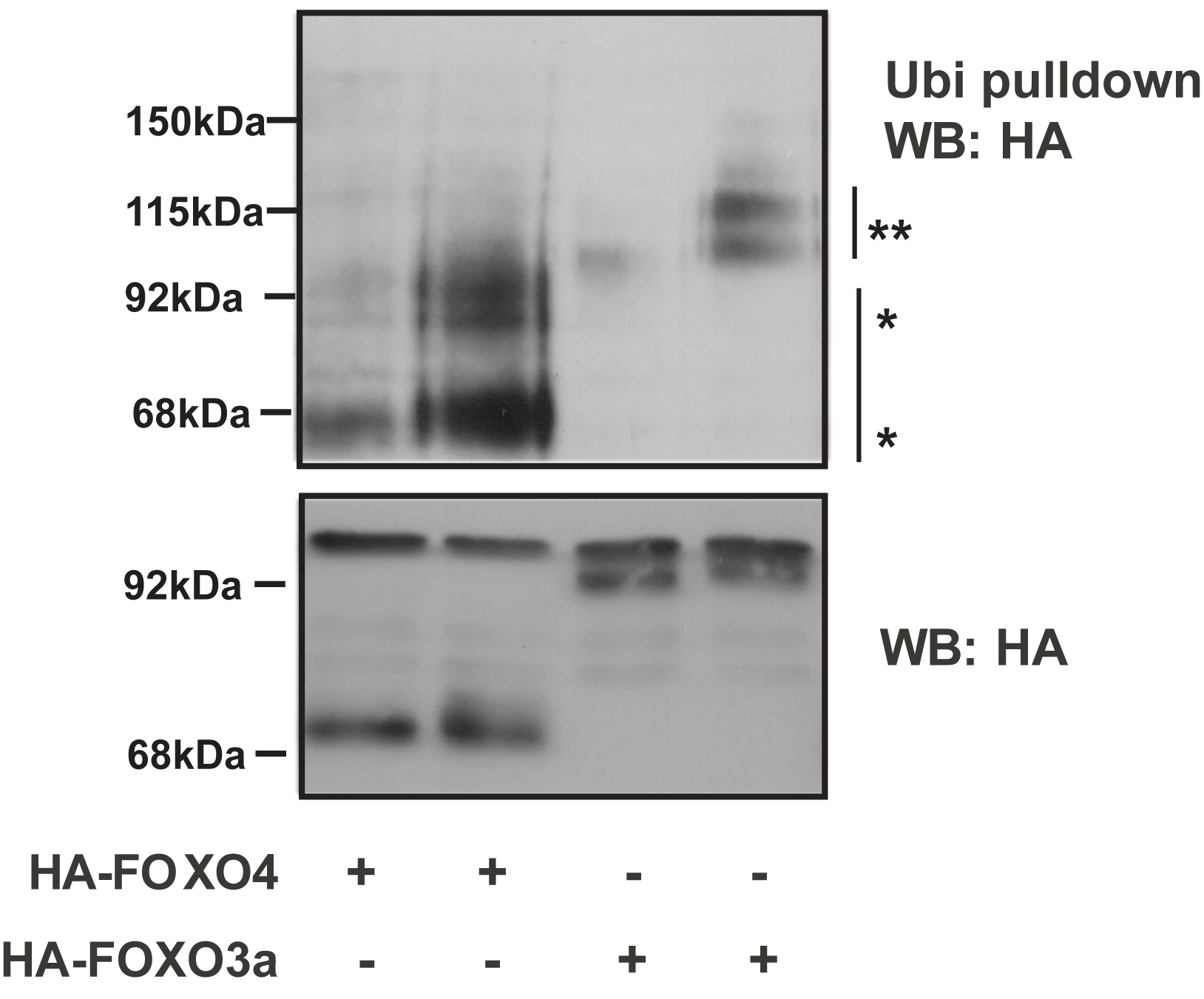

Supplement: Figure S3 — Mono-ubiquitination of FOXO4 and FOXO3a is induced upon peroxide stress. HEK293T cells were transfected with indicated constructs and His-Ubiquitin. Cells were left untreated or were treated with 50 µM H2O2 for 30 min, lysed and subjected to a ubiquitination assay. (*) Ubiquitinated FOXO4, (**) Ubiquitinated FOXO3a. (1.21 MB TIF) [file pone.0002819.s003.tif]
